# Towards integration of general practitioner posts and accident and emergency departments: a case study of two integrated emergency posts in the Netherlands

**DOI:** 10.1186/1472-6963-8-225

**Published:** 2008-11-04

**Authors:** Rudolf B Kool, Daniel J Homberg, Helen CM Kamphuis

**Affiliations:** 1 Quality and Safety Department, Prismant Institute for Research in Health Care, Papendorpseweg 65, 3528 BJ, Utrecht, the Netherlands

## Abstract

**Background:**

Accident and emergency (A&E) departments and general practitioner (GP) posts are often used inappropriately, leading to overcrowding. In the Netherlands, increasingly more integrated emergency posts (IEPs) are being created, integrating the care provided by GP posts and A&E departments, in order to improve the provision of the emergency care.

**Methods:**

This explorative study compares the efficiency and patient and employee satisfaction in IEPs with those in two GP posts and two A&E departments. To this end, information was retrieved from hospital and GP patient records for the first quarter of the year before and of the year after the creation of IEPs. Patients and employees were sent a questionnaire to measure their satisfaction. Lastly, groups of hospital doctors, GPs, GP assistants, and nurses were interviewed.

**Results:**

After the creation of IEPs, there was a shift of more than fifteen percent from secondary care to primary care for emergency consultations and waiting/consultation times were shortened by more than ten percent. Compared with the control settings, patients were more satisfied about telephone contact with an IEP, but professionals working at the IEP were less satisfied with several aspects of their work.

**Conclusion:**

IEPs could be a promising innovation to organize emergency care more efficiently; however, it might take time to convince professionals of the possible advantages. Studies involving more IEPs and longer follow-up times are needed to determine whether such integration should be stimulated.

## Background

In many countries accident and emergency (A&E) departments have a problem with inefficient patient flow because of overcrowding [1-8]. An important factor contributing to this overcrowding is self-referral: increasingly more patients go directly to A&E departments for out-of-hours services without first contacting their general practitioner (GP). At the same time, most of the problems seen in A&E departments are in principal primary care problems and can be regarded as inappropriate use of A&E services. Various studies report that between 15% and 50% of patients make inappropriate use of A&E services [9-18]. Most of these patients can be treated by a GP or nurse and at lower cost [19-26].

In the Netherlands, some GP posts were set up in the vicinity of a hospital or in the hospital itself, in an attempt to reduce self-referrals and inappropriate use of hospital services [27,28]. More recently, a GP post and a hospital in two regions (Purmerend and Haarlem) took a next step to optimize patient flow at local A&E departments by integrating the care provided by A&E departments and GP posts, forming so-called integrated emergency posts (IEPs). The purpose of these posts is to provide appropriate treatment within one organisation with complex, specialized care being provided by A&E doctors and less complex care being provided by GPs or specially trained nurses, such as nurse practitioners or physician assistants. In this way, it is expected that IEPs will shift the provision of care for certain health problems back from secondary care to primary care while providing patients with appropriate care for their problems.

To date (August 2008), seven IEPs have been established in the Netherlands and in several other regions A&E departments and GP posts are preparing to integrate their services. As similar innovations have not been described in the literature, we investigated whether IEPs meet expectations. To this end, we used data from the IEPs in Purmerend and Haarlem and two control locations to determine (i) whether an IEP changes patient flow by reducing the number of patients seen by A&E staff and by increasing the number of patients seen by the GP or nurse; (ii) whether waiting/consultation times are shorter, thereby speeding up the flow of patients through emergency services; (iii) whether patients treated at an IEP are more satisfied than patients treated at a GP post or A&E department; and (iv) whether doctors, nurses, and other professionals are satisfied with the concept of an IEP.

## Methods

### Sites

This study compares two IEPs with two comparable control settings, namely, traditional GP posts and A&E departments. In these control settings, patients with health problems occurring out of hours decide themselves whether to phone their GP or to visit the local A&E department. Patients phoning a GP post undergo triage by a GP assistant according to a protocol set up by the Dutch Association of General Practitioners. This triage is supervised by a GP. Possible outcomes of this triage are: self-care advice, advice to visit the own GP the next day, advice to visit the A&E department at the hospital, advice to visit the GP post to be seen by a GP or to been seen at home by a GP. Patients who come directly to a GP post are seen by the GP after registration and triage by the GP assistant. In the A&E department, a resident sees referred as well as self-referred patients after triage by an A&E nurse. Most Dutch A&E departments use the Manchester Triage System [29,30].

When the IEPs were set up, patients were informed about the changes to emergency care provision by means of leaflets, posters, and media coverage. At the IEP, patients check in at the joint reception of the GP post and the A&E department or call one telephone number. Triage is performed by a trained GP assistant (Purmerend) or nurse (Haarlem), using a standard protocol. She or he allocates the patient either to the A&E doctor, the GP or a nurse specially trained to treat simple pathology or to give self-care advice. A specially trained GP supervises the nurses and GP assistants. Table 1 gives an overview of the number of professionals working at the IEPs and the control sites.

**Table 1:** Numbers of staff working at the IEPs and control locations

	**IEP 1**		**Control 1**		**IEP 2**		**Control 2**	
	*5–11 PM*	*11 PM–8 AM*	*5–11 PM*	*11 PM–8 AM*	*5–11 PM*	*11 PM–8 AM*	*5–11 PM*	*11 PM–8 AM*
GP assistants	3	2	2–4	2	4–5	2	3	2
GPs	2	1	2–3	1	2–3	1	1–2	1
A&E doctors	1	1	1	1	1–2	1	1	1
Nurses	4–5	3	3	2	3–4	2	2–3	2

In some cases the number depends on the day of the week.

Control sites had to meet the following inclusion criteria: comparable number of patients visiting the A&E department and GP post annually, comparable urbanization and proportion of immigrants in catchment area and comparable healthcare facilities in the direct environment. Major changes in the cooperation between the GP post and the hospital were considered an exclusion criterion. On the basis of these criteria, we selected two combinations of a GP post and A&E department in Lelystad and Zaandam. Both cooperated with the study. Table 2 shows the characteristics of these sites.

**Table 2:** Characteristics of the settings (2006)

	**IEP 1**	**Control 1**	**IEP 2**	**Control 2**
Number of visits A&ED yearly	20–30.000	20–30.000	10–20.000	10.20.000
Number of contacts GP Post yearly	20–30.000	20–30.000	10–20.000	10.20.000
Other hospital within 10 km	No	No	No	No
Immigrants	10–20%	10–20%	10–20%	10–20%
Urbanization (inhabitants per km^2^)	> 2000	1000–2000	<500	<500

Sources: Central Office for Statistics, yearly reports hospitals and GP posts

### Outcome measures

The outcome measures for patient flow were the total number of patients seen out of hours at the A&E department, the GP post, or the IEP, time spent at the post, and percentage of self-referrals. Useful information about diagnosis was not available in the systems of the hospitals and this information was not reliable in the systems of the GP posts.

The outcome measures for patient satisfaction were satisfaction about accessibility of the location, waiting time, reception, interpretation of the problem, treatment and information provided, autonomy, discharge and aftercare. The outcome measures for employee satisfaction were satisfaction regarding autonomy, clarity about their tasks, staffing, patient care, use of personal capacities, social climate, information, culture, work, and organization.

### Design and data collection

Two IEPs (Purmerend and Haarlem) and two control sites (Lelystad and Zaandam) provided outcome information at T^0^, before the establishment of IEPs (Purmerend May 2005, Haarlem June 2006), and at T^1^, after the IEPs were established. The two IEPs recorded whether patients were seen by a GP/GP assistant or by an A&E doctor/nurse. Table 3 gives an overview of the moments of data collection for the four sites.

**Table 3:** Timetable for measurement of outcomes

	**IEP 1**		**Control 1**		**IEP 2**		**Control 2**	
	**T**^0^	**T**^1^	**T**^0^	**T**^1^	**T**^0^	**T**^1^	**T**^0^	**T**^1^
Patient flow	Q1 2005	Q1 2006	Q1 2005	Q1 2006	Q1 2006	Q1 2007	Q1 2006	Q1 2007
Patient satisfaction	No	May 2007	No	May 2007	No	May 2007	No	May 2007
Employee Satisfaction	No	May 2007	No	May 2007	No	May 2007	No	May 2007

#### Patient flow

At T^0 ^retrospective data were retrieved from the information systems of the hospital and the GP post for patients seen Monday to Friday between 5 pm and 8 am and on Saturday and Sunday in the first quarter of the year in which the IEP started and at T^1 ^in the first quarter of the year after the IEP was established. The same quarters were chosen to avoid seasonal variation.

#### Patient satisfaction

Patient satisfaction was measured with a specially designed questionnaire based on a validated survey used in most Dutch hospitals [31] but adjusted for an IEP setting. A questionnaire was also designed for patients contacting the settings by phone. The survey for patients visiting the IEP, GP post or A&E department, contained 45 questions that measured eight dimensions. The survey for patients contacting the IEP or GP post by phone, contained 27 questions that measured four dimensions. The patient satisfaction questionnaires were closed-ended questionnaires. Responses were given on a 5-point satisfaction scale ranging from "unsatisfied (1)" to "very satisfied (5)". The dimension scores were calculated as the mean item score. There was no retrospective information available about patient satisfaction.

The questionnaires were sent in May 2007 to the first 480 patients who had visited the IEP in a certain week and to the first 240 patients who had phoned the IEP. The same questionnaire was sent to the first 640 patients who had visited the control A&E departments and GP posts in the same week and to the first 160 patients who had phoned the control GP posts. After 10 days a reminder was sent. Questionnaires were returned by 151 patients who had visited the IEP (response 31%), by 102 patients who had phoned the IEP (response 43%), by 236 patients who had visited the control A&E departments and GP posts (response 37%), and by 57 patients who had phoned the GP Post (response 31%).

#### Employee satisfaction

Employee satisfaction was measured with a specially developed survey based on a validated questionnaire frequently used in Dutch hospitals [32] but adjusted for an IEP setting. Employee satisfaction was measured on ten dimensions. In total, there were 61 questions. The dimension scores were calculated as the mean item scores. Responses were given on a 5-point satisfaction scale ranging from "I totally do not agree (1)" to "I totally agree (5)". There was no retrospective information available about employee satisfaction.

All employees working at the IEP, A&E department, or GP post were sent a questionnaire in May 2007. Because the number of A&E doctors and GPs at the posts is flexible and not registered, the response rate is not known. In total, surveys were returned by 121 IEP employees and 103 employees working in the control settings. Further interviews were conducted with 44 randomly selected GPs, nurses, A&E doctors, and GP assistants working in the different settings to get a more complete picture of the process of integration and the views and emotions of the staff.

As we only used routinely administrative registered data and questionnaires for this observational study, approval of an ethics committee was not necessary in the Netherlands.

## Results

### Patient flow

In total 6257 patients visited the A&E department before the IEPs were established and 5715 were referred to an A&E doctor after the IEPs were established (Table 4). Over the same time period, the number of patients visiting the A&E departments in the control settings increased from 3985 to 4321. In total 10195 patients visited a GP post before the IEPs were established and 12940 were seen by a GP, GP assistant or nurse after the IEPs were established. In the control settings, the number of patients visiting a GP post decreased from 14011 to 12719.

**Table 4:** Patient flow in the IEP and control settings

	*IEP*		*Control*	
	*To*	*T1*	*To*	*T1*
Number of patients A&E	6257	5315**	3985	4321*
Number of contacts GP Post	10195	12940*	14011	12719**
Time spent at post	1 hr 56 min	1 hr 42 min**	1 hr 34 min	2 hrs*
% self-referrals	62%	46%**	53%	58%*

* T1 significantly higher, p < 0.05, Chi square ** T1 significantly lower, p < 0.05, Chi square

Waiting/consultation times decreased from 1 hour and 56 minutes before the IEPs were established to 1 hour 42 minutes after the IEPs were established. In the control settings, waiting/consultation times increased from 1 hour and 34 minutes to 2 hours. The proportion of self-referrals decreased from 62% before the IEPs were established to 46% after they were established. In the control settings, the proportion of self-referrals increased from 53% to 58%.

### Patient satisfaction

Patients who visited the IEPs were not more satisfied than those who visited the control GP posts or A&E departments (Table 5). However, patients who telephoned the IEP were more satisfied with the accessibility, interpretation of the question, and information provided than were patients who telephoned the control GP posts (Table 6). In the Netherlands, it is not possible to telephone A&E departments directly for triage and advice.

**Table 5:** Patient satisfaction with IEPs compared with the control setting

	**IEP**		**Control**	
	*n*	*mean*	*n*	*mean*
Accessibility	88	3.9	140	3.8
Waiting time	146	3.4	223	3.5
Reception	146	3.6	220	3.6
Information and communication	96	3.6	160	3.6
Autonomy	83	3.6	157	3.6
Discharge and aftercare	123	3.7	188	3.7
Interpretation of the question	135	3.7	216	3.8
Treatment	110	3.7	201	3.8

(1 = not satisfied at all, 5 = very much satisfied)

**Table 6:** Patient satisfaction with telephone contact with the IEPs compared with the control GP Post

	**IEP**		**Control GP Posts**	
	*n*	*mean*	*n*	*mean*
Accessibility	96	4.2*	53	3.9
Interpretation of the question	95	4.2*	52	3.9
Information and communication	91	3.7*	50	2.9
Discharge and aftercare	22	3.7	13	3.8

* significantly higher (p < 0.05), one-way ANOVA

(1 = not satisfied at all, 5 = very much satisfied)

### Employee satisfaction

Employees working at the IEPs were less satisfied than their colleagues working at GP posts or A&E departments regarding their autonomy, the social climate, the information provided by the organization, the culture of the organization, their satisfaction with their work, and the possibility to use their own capacities and skills (see Table 7).

**Table 7:** Satisfaction of employees working in an IEP or control setting

	**IEP**		**Control**	
	*n*	*mean*	*n*	*mean*
Autonomy	120	3.7	103	3.9*
Clarity about task	120	4.0	103	4.1
Staffing	120	3.1	103	3.0
Patient care	120	3.5	103	3.6
Social climate	120	3.5	103	3.8*
Information	120	3.2	103	3.4*
Culture	120	2.6	103	4.0*
Work	120	3.2	103	3.4*
Organization	120	4.0	103	4.2
Use of personal capacities	120	3.2	103	3.4*

* significantly higher (p < 0.05), one-way ANOVA

(1 = not satisfied at all, 5 = very much satisfied)

### Interviews

Several conclusions could be drawn from the interviews. While almost all staff supported the idea of an IEP, they had problems with realizing the concept in practice. Several nurses and GP assistants were not satisfied about their increased responsibility; they did not choose this new profession, and several conditions such as a solid triage system were not fulfilled. Lastly, staff mentioned practical problems such as the GP post and the A&E department still having separate meeting rooms, which did not facilitate integration.

## Discussion

This study showed that the development of an IEP leads to a shift from secondary care to primary care, a decrease in waiting/consultation times, a decrease in self-referrals, and increased patient satisfaction with telephone contact with care providers. There was no difference in satisfaction between patients who visited an IEP and those who visited a GP post or A&E department. In contrast, employees working at an IEP were less satisfied about their work and working conditions than employees working at GP posts or A&E departments.

However, it should be borne in mind that the integration of GP and hospital cultures and working style will take time, probably longer than the twelve months we allowed. Moreover, we collected data from only two locations, which limits the statistical power of this explorative research. The study had potential methodological limitations: the retrospective case-control design carried a risk of confounding bias, selection bias, and information bias. These risks were minimized by careful selection of the control locations and protocols for uniform data collection. And finally, the response rates to the patient satisfaction surveys were lower than in most patient satisfaction surveys in the Netherlands (40–60%). This restricts generalization for this part of our study.

The shift in patient flow was not caused by a change in patient population because patient characteristics (age, sex, and urgency of the health problem) were similar at T^0 ^and T^1^. The decrease in the number of patients seen by A&E doctors at the IEP could have been caused by regional developments, such as negative publicity about the hospital and/or its A&E department, changes in cooperation with ambulance services, or management policy decisions. However, no evidence for such problems was found during the interviews or during contact with the different sites.

Primary care costs less than hospital care, so the national introduction of IEPs could reduce national spending on health care. This aspect was not investigated in this study but it would be interesting to analyse the consequences of setting up IEPs on healthcare costs. On one hand, healthcare costs will be diminished by the shift from hospital care to primary care but on the other, the setting up of IEPs will require investment in staff training and numbers, and the provision of joint facilities, such as one check-in counter, one call-centre, and a meeting room. The latter may be necessary to bridge cultural differences between GP post employees and hospital employees.

The decrease in the number of self-referrals to A&E departments after the IEPs were established was expected because a substantial proportion of self-referred patients with minor complaints were sent to the GP after triage and registered in the information system of the GP post.

The decrease in waiting/consultation times at the IEP compared with GP posts or A&E departments is remarkable and could be due to improved triage and patient allocation in the IEPs, so that patients were seen by the most appropriate doctor. Other possible explanations for this decrease, such as changed patient characteristics and staff changes, are not likely since both were relatively stable during the study.

It is surprising that patients were not more satisfied with the IEP provision of care. Theoretically, setting up an IEP creates transparency; patients no longer have to decide whether to visit a GP or an A&E doctor. Moreover, waiting/consultation times were shortened. This lack of satisfaction might be because most patients were seen by nurses rather than by doctors, and the single check-in desk may have comprised patient privacy. Another reason could be that most patients had not recently visited an emergency service and therefore could not compare their experiences with earlier ones in a traditional setting.

Employees at the IEPs were not satisfied about several aspects of their work. This is most likely due to the dramatic changes in work processes as a result of introducing new care professionals and a new triage protocol. Such changes can have considerable consequences. For example, nurses and GP assistants gained more autonomy and responsibility, but this was not always welcomed. Moreover, introduction of IEPs could have financial consequences for employees. In 2007, hospitals and their consultants were paid according to the number of patients seen at the A&E department, yet with the development of IEPs A&E doctors saw fewer patients, which could have had negative financial consequences. In one of the IEPs, the hospital and medical staff had an agreement with the health insurance company that their loss would be compensated. This was not the case in the other IEP.

The introduction of IEPs did not have consequences for the income of GPs or the GP posts.

Crucial to the success of IEPs is that patients are seen by an appropriate professional. This means that triage systems should be effective and preferably uniform. The two IEPs in this study used different triage systems, but a uniform triage system has recently been introduced for Dutch hospitals, GP services, ambulances, and mental health services and is currently being tested in four regions. If this so-called Dutch Uniform Triage System functions as expected, it will help IEPs achieve an efficient workflow and patient-centred care.

IEPs will not resolve all the problems of emergency services and may have some limitations. It will take many years to bridge the cultural gap between primary and secondary care professionals. Moreover, accessible, well-functioning IEPs may even create a demand for emergency services. In lowly populated regions, the provision of acute primary care by GPs might be more efficient than integrated care.

A prospective study with more IEPs and a longer follow-up is needed to determine whether IEPs are a welcome innovation that should be stimulated. That is why we are hoping to stimulate more hospitals and GP posts at home and abroad to establish IEPs. Only with more participants can we gather enough data to enable more definite conclusions to be drawn about this promising innovation.

## Conclusion

IEPs could make emergency care more efficient. Patients are more satisfied with some aspects than they are with traditional GP posts and A&E departments. However, professionals are not yet convinced about the advantages. Other GP posts and A&E departments in different countries should be encouraged to set up IEPs.

## Competing interests

The authors declare that they have no competing interests.

## Authors' contributions

RBK designed the study, interpreted the results and drafted the manuscript. DJH and HCMK performed the analysis, interpreted the results and contributed to the discussion. All authors reviewed and edited the manuscript for intellectual content.

## Pre-publication history

The pre-publication history for this paper can be accessed here:


